# Which Constituents Determine the Antioxidant Activity and Cytotoxicity of Garlic? Role of Organosulfur Compounds and Phenolics

**DOI:** 10.3390/ijms25158391

**Published:** 2024-08-01

**Authors:** Paulina Furdak, Grzegorz Bartosz, Izabela Sadowska-Bartosz

**Affiliations:** 1 Laboratory of Analytical Biochemistry, Institute of Food Technology and Nutrition, College of Natural Sciences, University of Rzeszow, 4 Zelwerowicza Street, 35-601 Rzeszow, Poland; paulinaf2@o2.pl (P.F.); gbartosz@ur.edu.pl (G.B.); 2 Doctoral School, University of Rzeszow, 16C Rejtana Street, 35-959 Rzeszow, Poland

**Keywords:** antioxidant, garlic, organosulfur compounds, PEO1 cells, phenolics, SKOV-3 cells

## Abstract

Garlic is a vegetable with numerous pro-health properties, showing high antioxidant capacity, and cytotoxicity for various malignant cells. The inhibition of cell proliferation by garlic is mainly attributed to the organosulfur compounds (OSCs), but it is far from obvious which constituents of garlic indeed participate in the antioxidant and cytotoxic action of garlic extracts. This study aimed to obtain insight into this question by examining the antioxidant activity and cytotoxicity of six OSCs and five phenolics present in garlic. Three common assays of antioxidant activity were employed (ABTS^●^ decolorization, DPPH^●^ decolorization, and FRAP). Cytotoxicity of both classes of compounds to PEO1 and SKOV-3 ovarian cancer cells, and MRC-5 fibroblasts was compared. Negligible antioxidant activities of the studied OSCs (alliin, allicin, *S*-allyl-D-cysteine, allyl sulfide, diallyl disulfide, and diallyl trisulfide) were observed, excluding the possibility of any significant contribution of these compounds to the total antioxidant capacity (TAC) of garlic extracts estimated by the commonly used reductive assays. Comparable cytotoxic activities of OSCs and phenolics (caffeic, *p*-coumaric, ferulic, gallic acids, and quercetin) indicate that both classes of compounds may contribute to the cytotoxic action of garlic.

## 1. Introduction

Garlic (*Allium sativum* L.) is a plant species widely used globally for culinary purposes as a spice but also as a prophylactic and a traditional remedy for diverse ailments [1,2]. It is the second most frequently utilized species within the *Allium* genus, following onions (*Allium cepa* L.) [3]. Garlic owes its popularity to numerous beneficial effects, including antioxidant [4,5,6], antiphlogistic [7], antidiabetic [8], antiatherosclerotic [9], antibacterial [10], and anticancer [7,11,12,13], as well as antifungal [14]. The first mention of the therapeutic use of garlic for curing tumors comes from 1550 BC, when Egyptians recognized its therapeutic benefits for various diseases and employed it both orally and topically [15].

The high content of antioxidants in garlic is well documented [4,5,6,16]. Garlic was found to have the highest oxygen radical absorbance capacity (ORAC) among over twenty popular vegetables, which evidences high activity of its components for scavenging peroxyl radicals [17].

Garlic is also an anticancer agent. The raw garlic extract was reported to hamper the growth of various malignant cells, showing the highest efficacy among 34 raw vegetable extracts, and exerting no discernible impact on nonmalignant cells [18]. Our previous study identified garlic extracts as the most cytotoxic to human ovarian cancer cells among extracts of 17 popular vegetables (including onion), fruits, and herb infusions [19]. Furthermore, garlic was also reported to have anticarcinogenic properties. Consistent consumption of garlic was evidenced to reduce the risk of incidence of cancer of the lung, breast, stomach, colon, and prostate [20].

It is generally assumed that the health-promoting attributes, including the anticancer activity and the characteristic smell of garlic, are mainly due to garlic organosulfur compounds (OSCs), among them allicin, allyl sulfide (AS), diallyl sulfide (DAS), diallyl disulfide (DADS), diallyl trisulfide (DATS), diallyl tetrasulfide (DATeS), dipropyl disulfide (DPDS), ajoene, allyl methyl thiosulfonate, 1-propenyl allyl thiosulfonate, L-glutamyl-*S*-alkyl-L-cysteine, *S*-allylcysteine (SAC), and *S*-allylmercaptocysteine (SAMC) [21,22,23]. Both raw and granulated forms of garlic are characterized by the prevalence of alliin and other odorless OSCs such as cysteine sulfoxides and isoalliin [24]. Upon crushing or mechanically damaging garlic, OSCs are liberated in the cytoplasm and the enzyme alliinase cleaving them is simultaneously liberated from the vacuoles. The reaction products are low-molecular-weight thiosulfinates, which rapidly undergo a series of nonenzymic reactions to yield a complex mixture of sulfur products, the composition of which changes over time [25]. Alliin is converted into allicin [*S*-(prop-2-en-1-yl)prop-2-ene-1-sulfinothioate], which constitutes about 60–70% of garlic OSCs formed during crushing of garlic cloves [26], and it is recognized as the most active garlic OSC [27,28].

Allicin hampered the growth of murine and human malignant cells and induced their apoptosis [29,30,31]. This compound prevented the induction of gastric cancer as well [32]. Apart from allicin, other OSCs including ajoene, DAS, DADS, DATS, DATeS, DPDS, SAC, and SAMC, were demonstrated to decrease the proliferation of various malignant cells, as reviewed in [13,33].

SAMC, administered intragastrically, inhibited the development of xenograft tumors formed by human HO8910 and SKOV-3 cells. SAC and SAMC inhibited the migration of SKOV-3 cells and their invasiveness [34]. Prolonged treatment with SAC inhibited proliferation and provoked apoptosis of A2780 human ovarian carcinoma cells and attenuated migration and invasiveness of these cells [35].

While it is generally assumed that OSCs condition the cytotoxicity of garlic to malignant cells, the determinants of the antioxidant capacity of garlic are less understood. When comparing the composition and TAC of various garlic cultivars, significant correlations were reported between the level of alliin and antioxidant capacity determined by the ABTS^•^ reduction, DPPH^•^ reduction, and FRAP methods [36]; nevertheless the comparison included only four cultivars, and the alliin content could be associated with the content of other compounds. Allicin was reported to act as a chain-breaking antioxidant [37], but its reaction rate constant with cumene peroxyl radicals hydroperoxide was rather low (2.6 × 10^3^ M^−1^ s^−1^) [38]. DADS was found to be an efficient lipid peroxidation terminator; nonetheless, alliin, allicin, and SAC were ineffective in the prevention of induced microsomal lipid peroxidation [39].

Garlic contains many other biologically active components beyond OSCs as well; among them are flavonoids and other phenolic compounds, steroids, saponins, polysaccharides, vitamins, and proteins, including lectins and enzymes. These constituents are likely to exhibit additive, synergistic, or antagonistic effects with OSCs [40,41,42].

Phenolics are known to be good antioxidants [43,44], but also anticancer agents [45,46], so they may be expected to have the cytotoxic effects of garlic. This study aimed to address this problem by examining the antioxidant activities of individual compounds belonging to the groups of OSCs and phenolics and their cytotoxic activities toward two ovarian cancer cell lines and human fibroblasts.

## 2. Results

**Figure 1:**
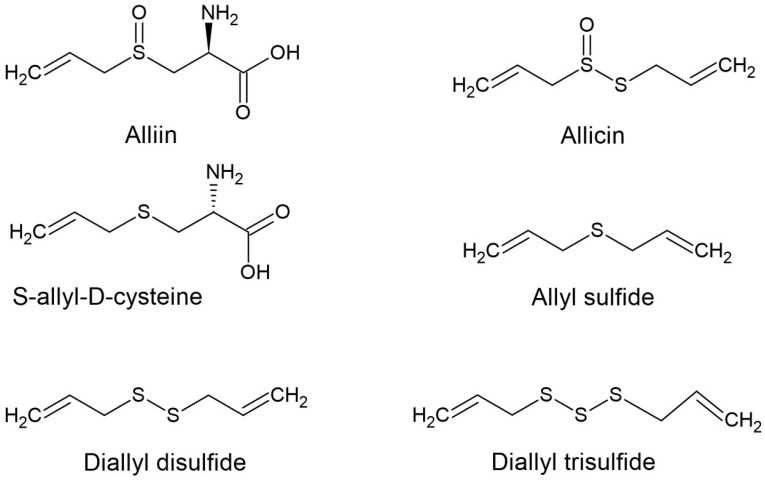
Structures of organosulfur compounds of garlic used in this study.

**Figure 2:**
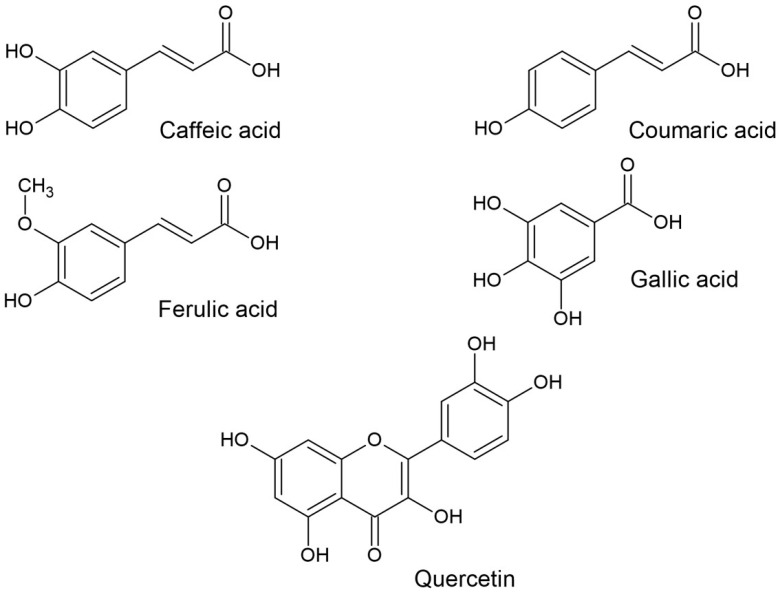
Structures of phenolic compounds of garlic used in this study.

The structures of the OSCs and phenolics used in this study are shown in Figure 1 and Figure 2, respectively.

A comparison of antioxidant properties of various garlic components demonstrated negligible antioxidant activities of OSCs studied in all antioxidant assays employed. In contrast, the studied phenolics showed considerable antioxidant activities (Table 1).

**Figure 3:**
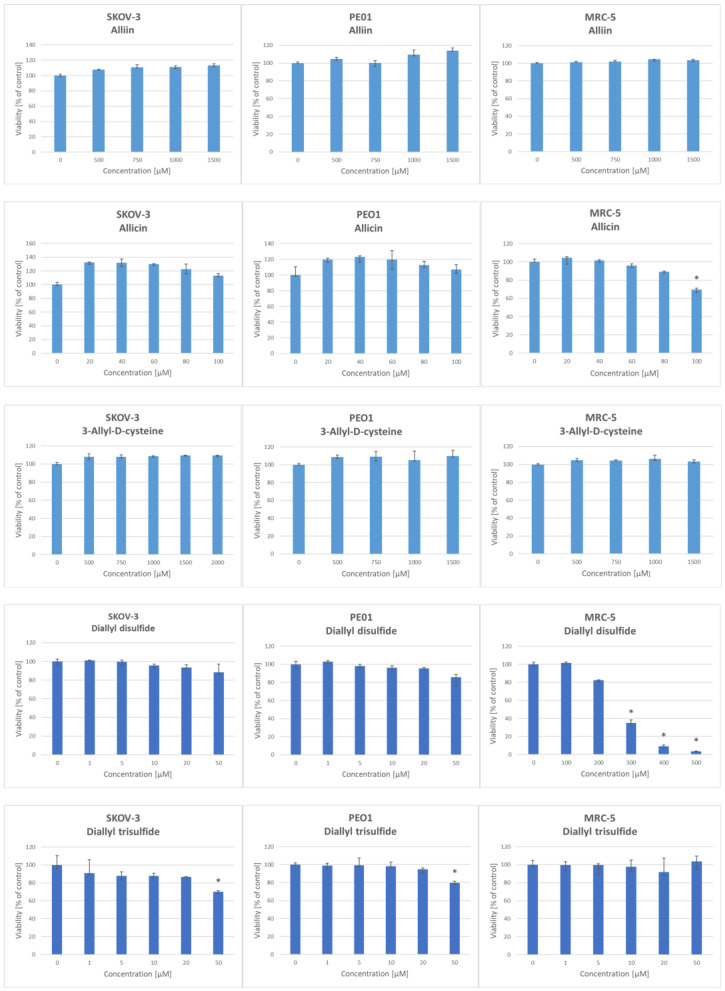
Effect of selected garlic organosulfur compounds on the survival of SKOV-3, PEO1, and MRC-5 cells. Exposure: 24 h; *n* ≥ 3; * *p* < 0.05.

In the cytotoxicity experiments, two human ovarian cancer cell lines (SKOV-3 and PEO1) and a normal human fibroblast MRC-5 cell line were used. The proliferation of ovarian cancer cells and fibroblasts was inhibited by compounds belonging to both groups. Somewhat surprisingly, the cytotoxic activities of OSCs were far from striking. Allin and *S*-allyl-D-cysteine did not significantly decrease the viability of PEO1, SKOV-3, and MRC-5 cells at concentrations between 500 and 1500 µM. Allicin decreased the viability of MRC-5 fibroblasts at a concentration of 100 µM; nevertheless, it did not significantly lower the viability of SKOV-3 and PEO1 ovarian cancer cells in the concentration range studied. Allyl sulfide did not decrease the viability of any tested cells at concentrations up to 100 µM, but at concentrations of 200 to 500 µM, it lowered the viability of SKOV-3 and MRC-5 cells. DADS significantly decreased the survival of MRC-5 cells only at a concentration of 300 µM. DATS did not change the viability of normal MRC-5 cells in the concentration range of 0 to 50 µM, but lowered the viability of SKOV-3 and PEO1 cells at the concentration of 50 µM (Figure 3).

**Figure 4:**
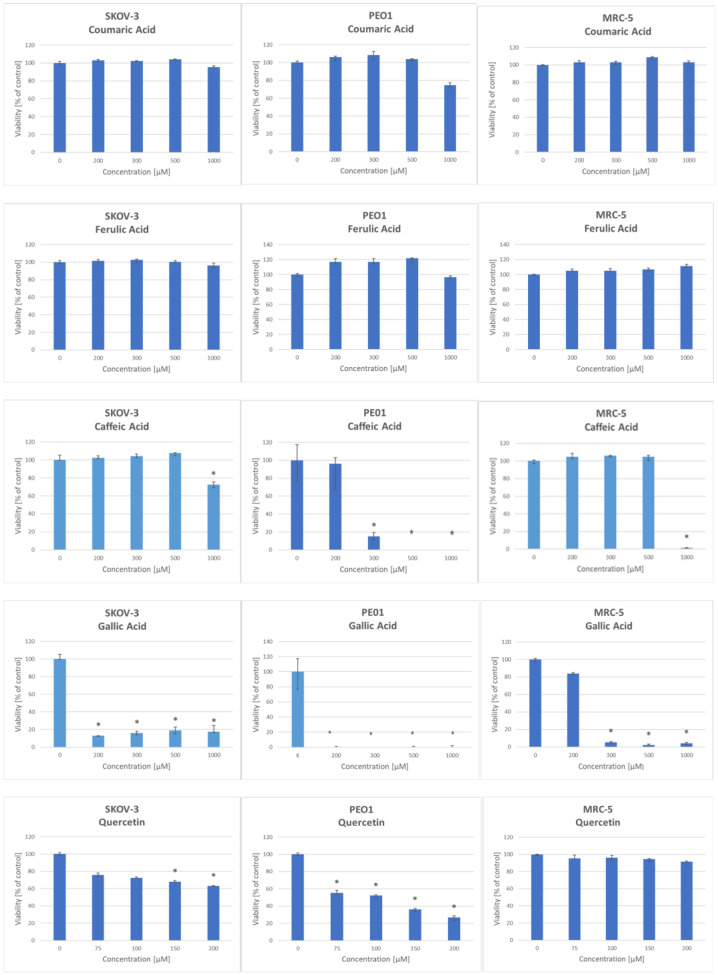
Effect of selected garlic phenolic compounds on the survival of SKOV-3, PEO1, and MRC-5 cells. Exposure: 24 h; *n* ≥ 3; * *p* < 0.05.

Among the phenolic compounds present in garlic, coumaric and ferulic acids did not cause any significant change in the viability of MRC-5, SKOV-3, or PEO1 cells, within the concentration range of 0–1000 µM. Caffeic acid decreased the viability of MRC-5 cells at a concentration of 1000 µM and of PEO1 ovarian cancer cells in the concentration range of 300–1000 µM. SKOV-3 cells were resistant to the effects of caffeic acid within the studied concentration range up to 1000 µM. Among the tested compounds, gallic acid exhibited the highest toxicity at concentrations of 200–1000 µM, reducing the viability of all cell lines studied. Quercetin did not decrease the viability of MRC-5 cells at concentrations up to 200 µM; nonetheless, it lowered the viability of SKOV-3 and PEO1 cells at concentrations of 75–200 µM (Figure 4). The concentrations of organosulfur compounds and phenolics lowering cell viability by 50% (IC_50_ values) are shown in Table 2.

## 3. Discussion

The presented results indicate the lack of reactivity of OSCs in standard antioxidant activity assays such as ABTS^●^ and DDPH^●^ decolorization assays and FRAP, based on the reduction in stable free radicals or Fe^3+^ ions. This finding is in line with the report of Mahmutovic et al. [47] on the lack of dependence between the TAC of bulbs and leaves of various samples of garlic and ramson, as well as their OSC content. Analysis of our previous results [48] points to a lack of significant correlations between the sum of organosulfur compounds and TAC estimated by ABTS^●^ and DDPH^●^ decolorization and FRAP assays (r of 0.22, 0.06, and −0.39, respectively; *n* = 6). In contrast, the phenolic compounds studied showed considerable antioxidant activity, indicating that compounds of this class contribute to the TAC of garlic extracts and, together with ascorbic acid, can be the main determinants of TAC measured by the reductive assays. Other studies reported TAC of garlic extracts to be well correlated with the total phenolic content [48,49,50]. High amounts of ascorbic acid were also found in garlic (0.16 to 0.34 mg/g in five Italian endemic varieties) [51].

Surprisingly, the cytotoxic effects of the studied compounds present in garlic, especially OSCs, found in this study were rather modest. The low cytotoxicities of OSCs in our study were partly due to our measuring the cytotoxic activity after 24 h incubation. Stronger effects are usually observed after 48 h or 72 h exposure, due to the trivial fact of proliferation of control cells, with which the amounts of treated cells are compared, during prolonged incubation. Nevertheless, much stronger cytotoxic activities were found by some authors, although the results of various studies vary markedly. The data cited below concern 24 h exposure unless stated otherwise; the values are often approximate and read from figures. Treatment with 50 nM allicin decreased human cervical squamous carcinoma SiHa cell viability to ca. 60% [52]. Survival of human gastric carcinoma AGS cells was diminished to ca. 50% by 10 μg/mL, i.e., about 62 μM allicin [53]. Allicin concentrations lowering the survival of MDA-MB-231 and MCF-7 breast cancer cells by 50%, corresponding to about 18 and about 7 μg/mL (about 111 and 43 μM), respectively [54]. The viability of SKOV-3 cells was reduced to 50% by allicin concentration of 70–80 μg/mL (0.43–0.49 mM) [55], of human U251 glioma cells by ca. 40 μg/mL allicin (247 μM) [18], and of U87MG human glioblastoma cells by ca. 90/mL μg (0.55 mM) allicin [56] (data for 24 h exposure). Only a ca. 25% decrease in the survival of SGC7901 human gastric adenocarcinoma cells by 120 μg/mL (i.e., ca. 0.74 mM) allicin was reported [57]. Allicin lowered the viability of breast cancer MCF-7 and HCC-70 cells to 50% at concentrations of ca. 15 μM and ca. 10 μM, respectively, while their viability was decreased down to ca. 75% and ca. 85%, respectively, by 1 mM alliin [58]. However, in our comparison of the cytotoxic properties of various garlic extracts [48], the IC_50_ values for decreasing cell viability correlated negatively with the sum of contents of organosulfur compounds determined, but the correlations coefficients were below the level of statistical significance (r of −0.44, −0.50, and −0.44 for SCOV-3, PEO1, and MRC-5 cells, respectively; *n* = 6). These results can suggest that also other components of garlic extracts contribute to their cytotoxicity.

The viability of human cervical epidermoid carcinoma Ca Ski cells was decreased to about 50% by ca. 25 μM DADS [59] and of breast cancer MCF-7 cells by 400 μM DADS [60]. Diallyl disulfide reduced the survival of prostatic carcinoma LNCaP cells by about 30% by 100 μM DADS [61] and the survival of lung cancer A549 cells by about 20% by 200 μM DADS [62]. The survival of colorectal cancer HCT-116 cells was decreased to ca. 80% by 400 μM DADS [63]. DADS at the concentration of 1.5 mM was reported to reduce the viability of MDA-MB-468 cells to about 65% and of human lung epithelial BEAS-2B cells to about 75% [64].

There may be another important factor contributing to the variability of results concerning the cytotoxicity of garlic OSCs. These compounds are reactive, especially with thiols, forming covalent *S*-allyl conjugates [65,66,67]. If OSCs are introduced to the medium before the addition of the medium to the cells, they may react with components of the medium, especially with proteins of the fetal serum, with the fraction of unreacted compound decreasing as a function of time before addition to the cells and penetration of the plasma membrane of the cells. Such detail as the exact time interval between the dilution of an OSC with the medium and contact of the medium with the cell is not reported in standard protocols and may differ between experimenters. Importantly, the lower cytotoxicity values may be more relevant since, in the body, OSCs released from garlic may have even more opportunities to react with body components before reaching target cells.

The phenolic compounds present in garlic have also significant cytotoxic properties. *p*-Coumaric acid decreased the proliferation of neuroblastoma N2a cells by 50% at the concentration of 104 μM after 24 h [68], of A375 and B16 melanocytes at the concentrations of 2.5 and 2.8 mM, respectively [69], and of colon carcinoma HT-29 cells and human colorectal carcinoma HCT-15 cells with at the concentrations of 1.4 and 1.6 mM, respectively, after 48 h exposure [70].

Ferulic acid was also reported to have cytotoxic action against malignant cells. The viability of MDA-MB-231 cells was decreased to about 75% by 100 μM ferulic acid [71], of PC-3 and LNCaP prostate cancer cells to 50% by 300 μM and 500 μM ferulic acid, respectively [72], and of osteosarcoma 143B and MG63 cells to 50% by 60 μM and 66 μM ferulic acid, respectively (after 48 h) [73].

Caffeic acid decreased the viability of HT-1080 fibrosarcoma cells at the concentration of 30 μM [74], of MDA-MB-231 cells at the concentration of 151 μM [75], and of human cervical cancer HeLa cells by 50% at the concentration of about 2 mM (after 24 h exposure) [76].

Gallic acid is also a compound strongly inhibiting the proliferation of malignant cells and is considered to be a potential anticancer agent [77]. The IC_50_ values of gallic acid for P388-D1 mouse lymphoid cancer cells, HL-60RG cells human promyelocytic leukemia cells, HeLa human epithelial carcinoma cells, dRLh-84 rat hepatoma cells, PLC/PRF/5 human hepatoma cells, and KB human epidermoid carcinoma cells were reported to be 4.8, 5.4, 6.1, 6.2, 6.6, and 13.2 μg/mL (27, 30, 34, 34, 37, and 73 μM), respectively [78], while other authors found IC_50_ values of gallic acid for HeLa and HTB-35 cervical cancer cells between 20 and 25 μg/mL (111–139 μM) [79]. The IC_50_ values at 48 h exposure were 16, 21, and 16 μg/mL (89, 117, and 89 μM) for DUI45, LNCaP, and PC-3 prostate cancer cells, respectively [80].

The IC_50_ values of quercetin for four gastric cancer cell lines (HGC-27, NUGC-2, MKN-28, and MKN-7 were found to be in the range of 32–55 μM [81], ca. 40 μM, ca. 40 μM for A549 human adenocarcinomic alveolar basal epithelial cells [82], and 35 μM and 20 μM for colorectal cancer CACO-2 cells and SW-620 cells, respectively [83]. The viability of MCF-7 cells was diminished to ca. 70% by 40 μg/mL (132 μM) quercetin [84] and that of breast cancer HCC1937 cells to ca. 40% by 100 μM quercetin [85].

The values reported in the literature and found in this study needed to decrease the viability of cancer cells are mostly too high to be obtained in vivo; moreover, colonic fermentation decreasing their content in the food and limiting intestinal absorption as well as metabolism, may decrease their bioavailability. It was found, e.g., that colonic fermentation of garlic results in a 44 and 41% loss of phenolics in fresh garlic and black garlic, respectively, and a 33% decrease in OSCs [86]. Nevertheless, higher concentrations of these compounds can be released from food products and encountered by cells of the digestive tract or during a topical treatment.

Comparable sensitivity of cancer cells to garlic OSCs and phenolics found in our study suggests that not only OSCs but both types of compounds may contribute to the anticancer activity of garlic. The efficiency of different types of garlic in preventing fibrosarcoma growth in mice was found to correlate with the contents of not only allicin but also phenolics and flavonoids [87]. The antiproliferative activity of extracts of several varieties of Italian garlic did not correlate with their content of OSCs, and it was supposed that the cytotoxicity of garlic can be ascribed to the synergistic action of many metabolites, including ascorbate [51]. The results presented in this study seem to support this view. Organosulfur compounds may be more important as they are present in higher amounts than phenolics, but the latter can also have a contribution to the cytotoxicity of garlic for cancer cells. Moreover, interactions between various garlic constituents may affect the cytotoxic effects of garlic extracts. This question deserves further studies.

## 4. Materials and Methods

### 4.1. Reagents, Disposables and Equipment

Allyl sulfide (CAS no. 10152-76-8; cat. no. HY-128447, purity: 98.45%), 3-allyl-D-cysteine (CAS no. 770742-93-3, cat. no. HY-W048286), allicin (diallyl thiosulfinate, CAS no. 539-86-6, cat. no. HY-N0315, purity: 97.36%), alliin (CAS no. 556-27-4, cat. no. HY-N0661, purity: 99.86%), diallyl trisulfide (CAS no. 2050-87-5; cat. no. HY-117235, purity: ≥95.0%), 2,2-diphenyl-1-picrylhydrazyl (DPPH) (CAS no. 1898-66-4, cat. no. HY-112053, purity ≥ 99.13%), and iron(III) chloride (FeCl_3_) (CAS no. 7705-08-0; cat. no. 451649; purity ≥ 99.99%) were provided by MedChemExpress (Monmouth Junction, NJ, USA), Coumaric acid (CAS no. 501-98-4, cat. no. C9008, purity ≥ 99%), ferulic acid (CAS no. 1135-24-6, cat. no. PHR1791), gallic acid (CAS no. 149-91-7, cat. no. 91215, purity ≥ 98%), and caffeic acid (CAS no. 331-39-5, cat. no. C0625, purity ≥ 98%) were obtained from Santa Cruz Biotechnology (Santa Cruz, CA, USA). Quercetin (CAS no. 117-39-5, cat. no. Q4951, purity ≥ 95%), diallyl disulfide (CAS no. 2179-57-9, cat. no. HY-W015635, purity: 99.36%), dimethyl sulfoxide (DMSO) (CAS no. 67-68-5, cat. no. D2438, anhydrous, purity ≥ 99.9%), (±)-6-hydroxy-2,5,7,8-tetramethylchromane-2-carboxylic acid (Trolox) (CAS no. 53188-07-1, cat. no. 238813, purity ≥ 97%), Folin–Ciocalteu’s phenol reagent (cat. no. F9252), 2,4,6-tris(2-pyridyl)-s-triazine (TPTZ, CAS no. 3682-35-7, cat. no. T1253; purity ≥ 98%), phosphate-buffered saline (PBS) (cat. no. PBS404.200), and Neutral Red (CAS no. 553-24-2; solution 0.33%, cat. no. N2889) were purchased from Merck (Poznań, Poland). Ethanol (CAS no. 64-17-5, cat. no. 396480111, purity ≥ 99.8%), methanol (CAS no. 67-56-1, cat. no. 6219900110, purity ≥ 99.9%), and sodium acetate anhydrous (CAS no. 127-09-3, cat. no. BN60/6191; purity ≥ 99%) were obtained from Avantor Performance Materials (Gliwice, Poland). 2,2-Azino-bis (3-ethylobenzthiazoline-6-sulfonic acid) (ABTS) (CAS no. 504-14-6, catalog no. 10102946001, purity ≥ 99) was purchased from Roche (Warsaw, Poland). Hydrochloric acid (CAS no. 7647-01-0; cat. no. 115752837, 35–38%) was provided by Chempur (Piekary Śląskie, Poland).

The SKOV-3 (HTB-77) and MRC-5 (CCL-171) cells were obtained from the American Type Culture Collection (ATCC; Gaithersburg, MD, USA). The PEO1 (10032308) cells were provided by the European Collection of Authenticated Cell Cultures (ECACC; Salisbury, UK). Dulbecco’s Modified Eagle Medium + GlutaMax (DMEM + GlutaMax) (catalog no. 21885-025), Dulbecco’s Modified Eagle Medium (DMEM) (cat. no. 12430-054), and Dulbecco’s Phosphate Buffered Saline (DPBS) (catalog no. 14040-117) were purchased from Thermofisher Scientific (Waltham, MA, USA). Phosphate-buffered saline (PBS) without Ca^2+^ and Mg^2+^ (catalog no. 02-023-1A), Trypsin-EDTA solution (10×) (catalog no. 03-051-5B), fetal bovine serum (catalog no. 04-001-1A), and penicillin–streptomycin solution (catalog no. 03-031-1B), 0.4% Trypan Blue solution (catalog no. T8154) were obtained from Biological Industries (Cromwell, CT, USA). Water was purified by the Milli-Q system (Millipore, Bedford, MA, USA).

Cell culture 75 cm^2^ flasks (T75; cat. no. 156499) were provided by Thermofisher Scientific (Waltham, MA, USA). Transparent 96-well culture plates (cat. no 655180) were obtained from Greiner (Kremsmünster, Austria). Other sterile cell culture materials were obtained from Nerbe (Winsen, Germany).

Stock solutions of allicin, AS, DADS, DATS, caffeic, coumaric, and ferulic and gallic acids, as well as quercetin were prepared in DMSO. The effects of the solvent (if any) were subtracted from the effects exerted by the solutions containing DMSO (in all cases at final not exceeding 0.2%). Stock solutions of alliin and *S*-allyl-D-cysteine were made in PBS and diluted with the cell culture media.

Absorbance measurements were performed in a Spark multimode microplate reader (Tecan Group Ltd., Männedorf, Switzerland) using transparent flat-bottom 96-well plates (cat. no. 655101) bought from Greiner (Kremsmünster, Austria).

### 4.2. Estimation of the Antioxidant Activity

#### 4.2.1. ABTS^●^ Reduction Assay

A modification [88] of the ABTS^●^ decolorization assay [89] was employed. Briefly, various volumes of solutions of the studied compounds were introduced to wells of a 96-well microplate, each prefilled 200 μL of ABTS^●^ solution. The stock ABTS^●^ solution was diluted with PBS so that 200 μL of the sample had an initial absorbance of 1.0 at 734 nm in a well of a 96-well microplate. The drop in absorbance after 30 min incubation at room temperature was read as a measure of the antioxidant activity.

#### 4.2.2. DPPH^●^ Reduction Assay

The assay was performed as described in a previous article [90]. Briefly, increasing volumes of solutions of the studied compounds were introduced to wells of a 96-well microplate, each with 200 μL of 0.3 mM DPPH^●^ solution in methanol. The reaction was allowed to proceed for 30 min at ambient temperature in the dark, and absorbance at 517 nm was read. The absorbance decrease was a measure of the antioxidant activity.

#### 4.2.3. The Ferric Reducing Antioxidant Power (FRAP) Assay

The method proposed by Benzie and Strain [91] was employed with a slight modification. Briefly, increasing volumes of solutions of the studied compounds were added to wells of a 96-well microplate prefilled with 200 μL of the working solution composed of 0.3 M acetate buffer, pH 3.6 (10 volumes), 10 mM 2,4,6-tris(2-pyridyl)-s-triazine (TPTZ), in 40 mM HCl (1 volume) and 20 mM FeCl_3_ (1 volume), was prepared immediately before use. After 30 min incubation at ambient temperature and the absorbance of the Fe^2+^-TPTZ complex was read at 593 nm.

#### 4.2.4. Calculation of the Antioxidant Activity

All assays were standardized with respect to Trolox. The antioxidant activity was calculated in each case as a ratio of the slope of the dependence of absorbance change on the concentration of a studied compound to the slope of the dependence of absorbance change of standard samples concerning Trolox on the concentration of Trolox. Antioxidant activities were expressed in moles of Trolox equivalents (TE)/mol of a studied compound, as described previously [88].

### 4.3. Cell Culture

The PEO1 cells, SKOV-3 cells, and MRC-5 cells were grown at 37 °C and 5% CO_2_ in RPMI + GlutaMAX medium, McCoy’s 5A medium, and the DMEM + GlutaMAX medium, respectively. The media were supplemented with 10% heat-inactivated fetal calf serum and penicillin/streptomycin. The cell viability was evaluated using the Trypan Blue exclusion test. Cells were counted in a Thoma hemocytometer (Superior Marienfeld, Lauda-Königshofen, Germany).

In the cytotoxicity experiments, cells were grown in wells of sterile 96-well plates. The seeding density was 1 × 10^4^ for SKOV-3 and MRC-5 cells and 1.5 × 10^4^ for PEO1 cells. The cells were allowed to grow in the incubator for 24 h. Then, the medium was removed and new medium with an examined compound was added. The stock solutions of the compounds studied diluted with the appropriate cell medium were filtered before the addition to the cells using 0.22 µm filters. No more than 0.02% of DMSO was present in the media, and it had no discernible impact on the treated cells. Cells not exposed to any compound served as a control. After another 24 h incubation, the medium was gently aspirated, and the wells were added with 2% sterile Neutral Red solution (100 µL). The plates were then incubated (37 °C, 5% CO_2_) for one hour. The Neutral Red solution was aspirated, and the wells were washed two times with warm (37 °C) PBS. Subsequently, 100 µL of a permeabilization mixture (water/ethanol/glacial acetic acid, 50/49/1, *v*/*v*/*v*) was introduced to each well. The plates were shaken at 700 rpm for 20 min and absorbance was read at a wavelength of 540 nm against 620 nm.

### 4.4. Statistical Analysis

To assess the variances of differences between control cells and cells treated with appropriate compounds, the Kruskal–Wallis test was conducted (*n* ≥ 6 biological replicates), assuming differences with *p* ≤ 0.05 as statistically significant. The statistical analysis was carried out using the STATISTICA software package (version 13.1, StatSoft Inc., 2016, Tulsa, OK, USA).

## 5. Conclusions

Garlic organosulfur compounds show negligible activity in the common reductive assays of antioxidant activity (ABTS^●^ and DPPH^●^ decolorization assays and FRAP), so they cannot contribute significantly to the antioxidant capacity of garlic extracts measured by these assays. Apart from OSCs, phenolics inhibit cell proliferation and can contribute to the cytotoxicity of garlic.

## Figures and Tables

**Table 1:** Antioxidant activities of selected garlic components (in mol Trolox equivalents (TE)/mol compound).

Compound	ABTS^●^ Decolorization	DPPH^●^ Decolorization	FRAP
Organosulfur compounds			
Alliin	0.035 ± 0.012	0.004 ± 0.001	0.0006 ± 0.0007
Allicin	−0.038 ± 0.020	−0.023 ± 0.009	−0.0014 ± 0.0016
*S*-Allyl-D-cysteine	0.070 ± 0.059	−0.020 ± 0.004	0.0003 ± 0.0002
Allyl sulfide	0.016 ± 0.010	−0.006 ± 0.011	0.0026 ± 0.0010
Diallyl disulfide	0.013 ± 0.006	0.049 ± 0.014	0.0025 ± 0.0011
Diallyl trisulfide	0.030 ± 0.006	0.005 ± 0.0003	0.0022 ± 0.0004
Phenolics			
Caffeic acid	0.771 ± 0.066	0.001 ± 0.006	0.019 ± 0.002
Coumaric acid	0.585 ± 0.082	0.076 ± 0.005	0.153 ± 0.003
Ferulic acid	1.409 ± 0.096	0.185 ± 0.070	0.554 ± 0.017
Gallic acid	0.972 ± 0.028	0.178 ± 0.023	0.332 ± 0.001
Quercetin	1.138 ± 0.029	0.384 ± 0.040	1.350 ± 0.201

**Table 2:** IC_50_ values of selected garlic organosulfur compounds and phenolics for SKOV-3, PEO1, and MRC-5 cells.

Compound		IC_50_ [μM]	
	SKOV-3	PEO1	MRC-5
Organosulfur compounds			
Alliin	nd	263	321
Allicin	236	282	120
*S*-Allyl-D-cysteine	421	nd	578
Allyl sulfide	243	257	163
Diallyl disulfide	nd	265	268
Diallyl trisulfide	154	721	nd
Phenolics			
Caffeic acid	1317	245	nd
Coumaric acid	nd	1427	nd
Ferulic acid	nd	nd	nd
Gallic acid	115	19	243
Quercetin	272	100	111

Note: nd, determination of IC_50_ not possible on the basis of obtained data.

## Data Availability

Data available on request.

## References

[1] Espinoza T., Valencia E., Albarrán M., Díaz D., Quevedo R.A., Díaz O., Bastías J. (2020). Garlic (*Allium sativum* L.) and its beneficial properties for health: A review. Agroind. Sci..

[2] Melguizo-Rodríguez L., García-Recio E., Ruiz C., De Luna-Bertos E., Illescas-Montes R., Costela-Ruiz V.J. (2022). Biological properties and therapeutic applications of garlic and its components. Food Funct..

[3] Ekşi G., Özkan A.M.G., Koyuncu M. (2020). Garlic and onions: An eastern tale. J. Ethnopharmacol..

[4] Aydın S., Tahmas Kahyaoğlu D. (2020). Antioxidant effect potential of garlic in vitro and real food system: Effects of garlic supplementation on oxidation stability and sensory properties of butter. Eur. J. Lipid Sci. Technol..

[5] Farhat Z., Hershberger P.A., Freudenheim J.L., Mammen M.J., Hageman Blair R., Aga D.S., Mu L. (2021). Types of garlic and their anticancer and antioxidant activity: A review of the epidemiologic and experimental evidence. Eur. J. Nutr..

[6] Dinu M., Soare R., Băbeanu C., Botu M. (2023). Evaluation of productivity components and antioxidant activity of different types of garlic. Horticulturae.

[7] Stępień A.E., Trojniak J., Tabarkiewicz J. (2024). Anti-Cancer and anti-inflammatory properties of black garlic. Int. J. Mol. Sci..

[8] Saikat A.S.M., Hossain R., Mina F.B., Das S., Khan I.N., Mubarak M.S., Islam M.T. (2021). Antidiabetic effect of garlic. Rev. Farm..

[9] Li M., Yun W., Wang G., Li A., Gao J., He Q. (2022). Roles and mechanisms of garlic and its extracts on atherosclerosis: A review. Front. Pharmacol..

[10] Bhatwalkar S.B., Mondal R., Krishna S.B.N., Adam J.K., Govender P., Anupam R. (2021). Antibacterial properties of organosulfur compounds of garlic (*Allium sativum*). Front. Microbiol..

[11] De Greef D., Barton E.M., Sandberg E.N., Croley C.R., Pumarol J., Wong T.L., Das N., Bishayee A. (2021). Anticancer potential of garlic and its bioactive constituents: A systematic and comprehensive review. Sem. Cancer Biol..

[12] Catanzaro E., Canistro D., Pellicioni V., Vivarelli F., Fimognari C. (2022). Anticancer potential of allicin: A review. Pharmacol. Res..

[13] Mondal A., Banerjee S., Bose S., Mazumder S., Haber R.A., Farzaei M.H., Bishayee A. (2022). Garlic constituents for cancer prevention and therapy: From phytochemistry to novel formulations. Pharmacol. Res..

[14] Prajapati M., Shah M., Ranginwala A., Agrawal P., Acharya D., Thakkar S. (2021). Antifungal effects of tulsi, garlic, cinnamon and lemongrass in powder and oil form on *Candida albicans*: An in vitro study. J. Oral Maxillofac. Pathol..

[15] El-Bayoumy K., Sinha R., Pinto J.T., Rivlin R.S. (2006). Cancer chemoprevention by garlic and garlic-containing sulfur and selenium compounds. J. Nutr..

[16] Banerjee S.K., Mukherjee P.K., Maulik S.K. (2003). Garlic as an antioxidant: The good, the bad and the ugly. Phytother. Res..

[17] Fei M.L., Tong L.I., Wei L.I., De Yang L. (2015). Changes in antioxidant capacity, levels of soluble sugar, total polyphenol, organosulfur compound and constituents in garlic clove during storage. Ind. Crops Prod..

[18] Li C., Jing H., Ma G., Liang P. (2018). Allicin induces apoptosis through activation of both intrinsic and extrinsic pathways in glioma cells. Mol. Med. Rep..

[19] Furdak P., Pieńkowska N., Bartosz G., Sadowska-Bartosz I. (2022). Extracts of common vegetables inhibit the growth of ovary cancer cells. Foods.

[20] Fleischauer A.T., Arab L. (2001). Garlic and cancer: A critical review of the epidemiologic literature. J. Nutr..

[21] Mathew B., Biju R. (2008). Neuroprotective effects of garlic a review. Libyan J. Med..

[22] El-Saber Batiha G., Beshbishy A.M., Wasef L.G., Elewa Y.H.A., Al-Sagan A.A., Abd El-Hack M.E., Taha A.E., Abd-Elhakim Y.M., Devkota H.P. (2020). Chemical constituents and pharmacological activities of garlic (*Allium sativum* L.): A review. Nutrients.

[23] Zhang Y., Liu X., Ruan J., Zhuang X., Zhang X., Li Z. (2020). Phytochemicals of garlic: Promising candidates for cancer therapy. Biomed. Pharmacother..

[24] Borlinghaus J., Foerster Née Reiter J., Kappler U., Antelmann H., Noll U., Gruhlke M.C.H., Slusarenko A.J. (2021). Allicin, the odor of freshly crushed garlic: A review of recent progress in understanding allicin’s effects on cells. Molecules.

[25] Block E. (1992). The organosulfur chemistry of the genus *Allium*—implications for the organic chemistry of sulfur. Angew. Chemie Int. Edit..

[26] Han J., Lawson L., Han G., Han P. (1995). A spectrophotometric method for quantitative determination of allicin and total garlic thiosulfinates. Anal Biochem..

[27] Trio P.Z., You S., He X., He J., Sakao K., Hou D.-X. (2014). Chemopreventive functions and molecular mechanisms of garlic organosulfur compounds. Food Funct..

[28] Zeng Y., Li Y., Yang J., Pu X., Du J., Yang X., Yang T., Yang S. (2017). Therapeutic role of functional components in Alliums for preventive chronic disease in human being. Evid. Based Complement. Altern. Med..

[29] Hirsch K., Danilenko M., Giat J., Miron T., Rabinkov A., Wilchek M., Mirelman D., Levy J., Sharoni Y. (2000). Effect of purified allicin, the major ingredient of freshly crushed garlic, on cancer cell proliferation. Nutr. Cancer.

[30] Oommen S., Anto R.J., Srinivas G., Karunagaran D. (2004). Allicin (from garlic) induces caspase-mediated apoptosis in cancer cells. Eur. J. Pharmacol..

[31] Haghi A., Azimi H., Rahimi R. (2017). A comprehensive review on pharmacotherapeutics of three phytochemicals, curcumin, quercetin, and allicin, in the treatment of gastric cancer. J. Gastrointest. Cancer.

[32] Luo R., Fang D., Hang H., Tang Z. (2016). The mechanism in gastric cancer chemoprevention by allicin. Anti-Cancer Agents Med. Chem..

[33] Lanzotti V., Scala F., Bonanomi G. (2014). Compounds from *Allium* species with cytotoxic and antimicrobial activity. Phytochem. Rev..

[34] Chu Q., Ling M.T., Feng H., Cheung H.W., Tsao S.W., Wang X., Wong Y.C. (2006). A novel anticancer effect of garlic derivatives: Inhibition of cancer cell invasion through restoration of E-cadherin expression. Carcinogenesis.

[35] Xu Y.S., Feng J.G., Zhang D., Zhang B., Luo M., Su D., Lin N.M. (2014). *S*-allylcysteine, a garlic derivative, suppresses proliferation and induces apoptosis in human ovarian cancer cells in vitro. Acta Pharmacol. Sin..

[36] Soto V.C., Gonzalez R.E., Sance M.M., Galmarini C.R. Organosulfur and phenolic content of garlic (*Allium sativum* L.) and onion (*Allium cepa* L.) and its relationship with antioxidant activity. Proceedings of the VII International Symposium on Edible Alliaceae.

[37] Prasad K., Laxdal V.A., Yu M., Raney B.L. (1995). Antioxidant activity of allicin, an active principle in garlic. Mol. Cell. Biochem..

[38] Okada Y., Tanaka K., Sato E., Okajima H. (2006). Kinetic and mechanistic studies of allicin as an antioxidant. Org. Biomol. Chem..

[39] Chung L.Y. (2006). The antioxidant properties of garlic compounds: Allyl cysteine, alliin, allicin, and allyl disulfide. J. Med. Food.

[40] Li M., Yan Y.-X., Yu Q.-T., Deng Y., Wu D.-T., Wang Y., Zhao J. (2017). Comparison of immunomodulatory effects of fresh garlic and black garlic polysaccharides on RAW 264.7 macrophages. J. Food Sci..

[41] Sharma N. (2019). Efficacy of garlic and onion against virus. Int. J. Res. Pharm. Sci..

[42] Kovarovič J., Bystricka J., Vollmannová A., Tóth T., Brindza J. (2019). Biologically valuable substances in garlic (*Allium sativum* L.)—A review. J. Centr. Eur. Agric..

[43] Rasouli H., Farzaei M.H., Khodarahmi R. (2017). Polyphenols and their benefits: A review. Int. J. Food Propert..

[44] Swallah M.S., Sun H., Affoh R., Fu H., Yu H. (2020). Antioxidant potential overviews of secondary metabolites (polyphenols) in fruits. Int. J. Food Sci..

[45] Bhosale P.B., Ha S.E., Vetrivel P., Kim H.H., Kim S.M., Kim G.S. (2020). Functions of polyphenols and its anticancer properties in biomedical research: A narrative review. Translat. Cancer Res..

[46] Islam B.U., Suhail M., Khan M.K., Zughaibi T.A., Alserihi R.F., Zaidi S.K., Tabrez S. (2021). Polyphenols as anticancer agents: Toxicological concern to healthy cells. Phytother. Res..

[47] Mahmutovic O., Tahirovic I., Copra A., Memic M., Ibragic S., Karic L. (2014). Correlation of total secondary sulfur compounds, total phenols and antioxidant capacity in the Ramsons and Garlic. Br. J. Pharm. Res..

[48] Furdak P., Pieńkowska N., Kapusta I., Bartosz G., Sadowska-Bartosz I. (2023). Comparison of antioxidant and antiproliferative effects of various forms of garlic and ramsons. Molecules.

[49] Chen S., Shen X., Cheng S., Li P., Du J., Chang Y., Meng H. (2013). Evaluation of garlic cultivars for polyphenolic content and antioxidant properties. PLoS ONE.

[50] Škrovánková S., Mlček J., Snopek L., Planetová T. (2018). Polyphenols and antioxidant capacity in different types of garlic. Potrav. Slovak J. Food Sci..

[51] Fratianni F., Ombra M.N., Cozzolino A., Riccardi R., Spigno P., Tremonte P., Coppola R., Nazzaro F. (2016). Phenolic constituents, antioxidant, antimicrobial and anti-proliferative activities of different endemic Italian varieties of garlic (*Allium sativum* L.). J. Funct. Foods.

[52] Zhang Q., Yang D. (2019). Allicin suppresses the migration and invasion in cervical cancer cells mainly by inhibiting NRF2. Exp. Ther. Med..

[53] Park S.Y., Cho S.J., Kwon H.C., Lee K.R., Rhee D.K., Pyo S. (2005). Caspase-independent cell death by allicin in human epithelial carcinoma cells: Involvement of PKA. Cancer Lett..

[54] Maitisha G., Aimaiti M., An Z., Li X. (2021). Allicin induces cell cycle arrest and apoptosis of breast cancer cells in vitro via modulating the p53 pathway. Mol. Biol. Rep..

[55] Xu L., Yu J., Zhai D., Zhang D., Shen W., Bai L., Cai Z., Yu C. (2014). Role of JNK activation and mitochondrial bax translocation in allicin-induced apoptosis in human ovarian cancer SKOV3 cells. Evid. Based Complement. Alternat. Med..

[56] Cha J.H., Choi Y.J., Cha S.H., Choi C.H., Cho W.H. (2012). Allicin inhibits cell growth and induces apoptosis in U87MG human glioblastoma cells through an ERK-dependent pathway. Oncol. Rep..

[57] Zhang W., Ha M., Gong Y., Xu Y., Dong N., Yuan Y. (2010). Allicin induces apoptosis in gastric cancer cells through activation of both extrinsic and intrinsic pathways. Oncol. Rep..

[58] Rosas-González V.C., Téllez-Bañuelos M.C., Hernández-Flores G., Bravo-Cuellar A., Aguilar-Lemarroy A., Jave-Suárez L.F., Haramati J., Solorzano-Ibarra F., Ortiz-Lazareno P.C. (2020). Differential effects of alliin and allicin on apoptosis and senescence in luminal A and triple-negative breast cancer: Caspase, ΔΨm, and pro-apoptotic gene involvement. Fundam. Clin. Pharmacol..

[59] Lin Y.T., Yang J.S., Lin S.Y., Tan T.W., Ho C.C., Hsia T.C., Chiu T.H., Yu C.S., Lu H.F., Weng Y.S. (2008). Diallyl disulfide (DADS) induces apoptosis in human cervical cancer Ca Ski cells via reactive oxygen species and Ca^2+^-dependent mitochondria-dependent pathway. Anticancer. Res..

[60] Lei X.Y., Yao S.Q., Zu X.Y., Huang Z.X., Liu L.J., Zhong M., Zhu B.Y., Tang S.S., Liao D.F. (2008). Apoptosis induced by diallyl disulfide in human breast cancer cell line MCF-7. Acta Pharmacol. Sin..

[61] Gunadharini D.N., Arunkumar A., Krishnamoorthy G., Muthuvel R., Vijayababu M.R., Kanagaraj P., Srinivasan N., Aruldhas M.M., Arunakaran J. (2006). Antiproliferative effect of diallyl disulfide (DADS) on prostate cancer cell line LNCaP. Cell Biochem. Funct..

[62] Wu X.J., Kassie F., Mersch-Sundermann V. (2005). The role of reactive oxygen species (ROS) production on diallyl disulfide (DADS) induced apoptosis and cell cycle arrest in human A549 lung carcinoma cells. Mutat. Res..

[63] Song J.D., Lee S.K., Kim K.M., Park S.E., Park S.J., Kim K.H., Ahn S.C., Park Y.C. (2009). Molecular mechanism of diallyl disulfide in cell cycle arrest and apoptosis in HCT-116 colon cancer cells. J. Biochem. Mol. Toxicol..

[64] Sujatha P., Anantharaju P.G., Veeresh P.M., Dey S., Bovilla V.R., Madhunapantula S.R.V. (2017). Diallyl disulfide (DADS) retards the growth of breast cancer cells in vitro and in vivo through apoptosis induction. Biomed. Pharmacol. J..

[65] Rabinkov A., Miron T., Mirelman D., Wilchek M., Glozman S., Yavin E., Weiner L. (2000). S-Allylmercaptoglutathione: The reaction product of allicin with glutathione possesses SH-modifying and antioxidant properties. Biochim. Biophys. Acta Mol. Cell Res..

[66] Gruhlke M.C. (2019). Thiol-modification as important mode of action for allicin from garlic (*Allium sativum*). Multidisc. Digit. Publ. Inst. Proc..

[67] Furdak P., Bartosz G., Stefaniuk I., Cieniek B., Bieszczad-Bedrejczuk E., Soszyński M., Sadowska-Bartosz I. (2024). Effect of garlic extract on the erythrocyte as a simple model cell. Int. J. Mol. Sci..

[68] Shailasree S., Venkataramana M., Niranjana S.R., Prakash H.S. (2015). Cytotoxic effect of p-Coumaric acid on neuroblastoma, N2a cell via generation of reactive oxygen species leading to dysfunction of mitochondria inducing apoptosis and autophagy. Mol. Neurobiol..

[69] Hu X., Yang Z., Liu W., Pan Z., Zhang X., Li M., Liu X., Zheng Q., Li D. (2020). The anti-tumor effects of *p*-coumaric acid on melanoma A375 and B16 cells. Front. Oncol..

[70] Jaganathan S.K., Supriyanto E., Mandal M. (2013). Events associated with apoptotic effect of p-Coumaric acid in HCT-15 colon cancer cells. World J. Gastroenterol..

[71] Zhang X., Lin D., Jiang R., Li H., Wan J., Li H. (2016). Ferulic acid exerts antitumor activity and inhibits metastasis in breast cancer cells by regulating epithelial to mesenchymal transition. Oncol. Rep..

[72] Eroğlu C., Seçme M., Bağcı G., Dodurga Y. (2015). Assessment of the anticancer mechanism of ferulic acid via cell cycle and apoptotic pathways in human prostate cancer cell lines. Tumour Biol..

[73] Wang T., Gong X., Jiang R., Li H., Du W., Kuang G. (2016). Ferulic acid inhibits proliferation and promotes apoptosis via blockage of PI3K/Akt pathway in osteosarcoma cell. Am. J. Transl. Res..

[74] Rajendra Prasad N., Karthikeyan A., Karthikeyan S., Reddy B.V. (2011). Inhibitory effect of caffeic acid on cancer cell proliferation by oxidative mechanism in human HT-1080 fibrosarcoma cell line. Mol. Cell. Biochem..

[75] Kabała-Dzik A., Rzepecka-Stojko A., Kubina R., Jastrzębska-Stojko Ż., Stojko R., Wojtyczka R.D., Stojko J. (2017). Migration rate inhibition of breast cancer cells treated by caffeic acid and caffeic acid phenethyl ester: An in vitro comparison study. Nutrients.

[76] Chang W.C., Hsieh C.H., Hsiao M.W., Lin W.C., Hung Y.C., Ye J.C. (2010). Caffeic acid induces apoptosis in human cervical cancer cells through the mitochondrial pathway. Taiwan J. Obstet. Gynecol..

[77] Jiang Y., Pei J., Zheng Y., Miao Y.J., Duan B.Z., Huang L.F. (2022). Gallic acid: A potential anti-cancer agent. Chin. J. Integr. Med..

[78] Inoue M., Suzuki R., Sakaguchi N., Li Z., Takeda T., Ogihara Y., Jiang B.Y., Chen Y. (1995). Selective induction of cell death in cancer cells by gallic acid. Biol. Pharm. Bull..

[79] Zhao B., Hu M. (2013). Gallic acid reduces cell viability, proliferation, invasion and angiogenesis in human cervical cancer cells. Oncol. Lett..

[80] Chen H.M., Wu Y.C., Chia Y.C., Chang F.R., Hsu H.K., Hsieh Y.C., Chen C.C., Yuan S.S. (2009). Gallic acid, a major component of *Toona sinensis* leaf extracts, contains a ROS-mediated anti-cancer activity in human prostate cancer cells. Cancer Lett..

[81] Yoshida M., Sakai T., Hosokawa N., Marui N., Matsumoto K., Fujioka A., Nishino H., Aoike A. (1990). The effect of quercetin on cell cycle progression and growth of human gastric cancer cells. FEBS Lett..

[82] Nguyen T.T., Tran E., Nguyen T.H., Do P.T., Huynh T.H., Huynh H. (2004). The role of activated MEK-ERK pathway in quercetin-induced growth inhibition and apoptosis in A549 lung cancer cells. Carcinogenesis.

[83] Zhang X.A., Zhang S., Yin Q., Zhang J. (2015). Quercetin induces human colon cancer cells apoptosis by inhibiting the nuclear factor-kappa B Pathway. Pharmacogn. Mag..

[84] Deng X.H., Song H.Y., Zhou Y.F., Yuan G.Y., Zheng F.J. (2013). Effects of quercetin on the proliferation of breast cancer cells and expression of survivin in vitro. Exp. Ther. Med..

[85] Gulati N., Laudet B., Zohrabian V.M., Murali R.A.J., Jhanwar-Uniyal M.E.E.N.A. (2006). The antiproliferative effect of Quercetin in cancer cells is mediated via inhibition of the PI3K-Akt/PKB pathway. Anticancer Res..

[86] Moreno-Ortega A., Di Pede G., Pereira-Caro G., Calani L., Mena P., Del Rio D., Moreno-Rojas J.M. (2022). In vitro colonic fermentation of (poly)phenols and organosulfur compounds of fresh and black garlic. J. Agric. Food Chem..

[87] Shirzad H., Taji F., Rafieian-Kopaei M. (2011). Correlation between antioxidant activity of garlic extracts and WEHI-164 fibrosarcoma tumor growth in BALB/c mice. J. Med. Food.

[88] Kut K., Cieniek B., Stefaniuk I., Bartosz G., Sadowska-Bartosz I. (2022). A modification of the ABTS^•^ decolorization method and an insight into its mechanism. Processes.

[89] Re R., Pellegrini N., Proteggente A., Pannala A., Yang M., Rice-Evans C. (1999). Antioxidant activity applying an improved ABTS radical cation decolorization assay. Free Radic. Biol. Med..

[90] Kuczera K., Naparło K., Soszyński M., Bartosz G., Sadowska-Bartosz I. (2023). Capsaicin toxicity to the yeast *Saccharomyces cerevisiae* is not due to oxidative stress but to disruption of membrane structure. Chem. Biol. Interact..

[91] Benzie I.F., Strain J.J. (1996). The ferric reducing ability of plasma (FRAP) as a measure of “antioxidant power”: The FRAP assay. Anal. Biochem..

